# Supplementary material for: Knowledge of prevention of COVID-19 among the general people in Bangladesh: A cross-sectional study in Rajshahi district
Source: PLoS One. 2020 Dec 9;15(12):e0243410. doi: 10.1371/journal.pone.0243410 (PMC7725348; doi:10.1371/journal.pone.0243410)
Supplement: Attachment — Submitted filename: Response to editor and reviewer comments.docx [file pone.0243410.s002.docx]

**Response to editor and reviewer comments**

**Response to editor comments/ Journals requirements:**

1. It was written due to journal style.
2. All authors checked the manuscript thoroughly. Moreover, it was edited by Minara Jesmin, Graduate Teaching Partner, Hewens Primary School. Hayes,UB4 8JP, London, United Kingdom for helping grammatical correction of this study,
3. Data is available in the PLOS ONE journal website.

**Response to reviewer’s comments:**

1. Conclusions is revised as per reviewers-1 advice.
2. The statistical analysis has been used appropriately and rigorously according to the suggestions of reviewesrs-1.
3. Yes, data is available in the PLOS ONE journal website due to advice of reviewers-1.
4. Typographical or grammatical errors were corrected at revision by authors and Minara Jesmin, Graduate Teaching Partner, Hewens Primary School. Hayes,UB4 8JP, London, United Kingdom.

**Reviewer comment’s to the authors:**

**Reviewer#1:** The grammatical errors throughout the manuscript were corrected. The method sample size determination is revised. The word piloting omitted from the manuscript. It was noted that it was unfortunately stayed in the manuscript. Age is categories for better finding in the analysis of chi-square test and logistic regression analysis. Further study we will follow your advice. The knowledge on prevention of COVID-19 was assessed based on four components according to WHO and DGHS, Ministry of Health and Family Welfare, Government of Bangladesh**.** The conclusion is revised as per your mentorship. In future, we try to comprehensive study on COVID-19.

**Reviewer#2:**

**In Abstract:**

**In Background:** Issue is omitted

**In Methodology:** We used adult respondents

**In methodology:** Good knowledge indicates the respondents who answered four question yes.

**In results:** We write physical distancing instead of social distancing

**In introduction:**

**Line 64-69:** reference is cited

**Line 66:** The sentence is rewritten

**Line76:** This sentence is deleted

**Line 78:** We write another study instead of another article

**Line81:** The sentence is corrected

**Line 82:** We write COVID-19 instead of corona virus

**In Methods:**

**Line 102,107:** We write COVID-19 instead of corona virus

**Line 108-109:** The sentence is rewritten

**Line111:** We write not knowledgeable instead of no knowledgeable

**In Results:**

Duplication is removed

**Line 149-150:** The sentence is revised

Duplication is omitted

**In Discussion:**

The discussion section is rewritten due to suggestions of reviewer’s comments

Strengths and limitations are added in the manuscript

**In conclusion:**

The conclusion is revised according to reviewer’s suggestions
